# CAPICE: a computational method for Consequence-Agnostic Pathogenicity Interpretation of Clinical Exome variations

**DOI:** 10.1186/s13073-020-00775-w

**Published:** 2020-08-24

**Authors:** Shuang Li, K. Joeri van der Velde, Dick de Ridder, Aalt D. J. van Dijk, Dimitrios Soudis, Leslie R. Zwerwer, Patrick Deelen, Dennis Hendriksen, Bart Charbon, Marielle E. van Gijn, Kristin Abbott, Birgit Sikkema-Raddatz, Cleo C. van Diemen, Wilhelmina S. Kerstjens-Frederikse, Richard J. Sinke, Morris A. Swertz

**Affiliations:** 1 Department of Genetics, University of Groningen, University Medical Center Groningen, Groningen, the Netherlands; 2 Genomics Coordination Center, University of Groningen, University Medical Center Groningen, Groningen, the Netherlands; 3 Bioinformatics Group, Wageningen University & Research, Wageningen, the Netherlands; 4 Biometris, Wageningen University & Research, Wageningen, the Netherlands; 5 Donald Smits Center for Information and Technology, University of Groningen, Groningen, the Netherlands

**Keywords:** Variant pathogenicity prediction, Machine learning, Exome sequencing, Molecular consequence, Allele frequency, Clinical genetics, Genome diagnostics

## Abstract

Exome sequencing is now mainstream in clinical practice. However, identification of pathogenic Mendelian variants remains time-consuming, in part, because the limited accuracy of current computational prediction methods requires manual classification by experts. Here we introduce CAPICE, a new machine-learning-based method for prioritizing pathogenic variants, including SNVs and short InDels. CAPICE outperforms the best general (CADD, GAVIN) and consequence-type-specific (REVEL, ClinPred) computational prediction methods, for both rare and ultra-rare variants. CAPICE is easily added to diagnostic pipelines as pre-computed score file or command-line software, or using online MOLGENIS web service with API. Download CAPICE for free and open-source (LGPLv3) at https://github.com/molgenis/capice.

## Background

The past decades have seen rapid advances in genetic testing and increasing numbers of trial studies aimed at using genetic testing to facilitate rare disease diagnostics, and many studies have now demonstrated the unique role whole exome and genome sequencing can play in improving diagnostic yield [1–7]. However, the vast amount of genomic data that is now available has created large interpretation challenges that can be alleviated using computational tools. Nonetheless, variant interpretation in particular still remains time-consuming, in part because of the limited accuracy of current computational prediction methods and the manual work required to identify large numbers of false positives produced by those methods [8–10].

Existing prediction methods can be categorized into two groups. One group of methods [11, 12] focuses on specific types of variants. The majority of these methods can only classify non-synonymous single nucleotide variants (nsSNVs) [13, 14]. Successful methods of this group include Clinpred [15], which has the best current performance validated in multiple datasets, and REVEL [16], which specifically targets rare variants. However, these methods cannot give pathogenicity predictions and, hence, may miss the diagnosis when the causal variant is not an nsSNV, which is the case for 76% of reported pathogenic variants [17]. The other category of prediction methods provides predictions of selective constraints without the limitation of nsSNVs. However, they only indirectly predict the variant pathogenicity, using selective constraints to indicate the pathogenicity [18–21]. A method that is widely used and acknowledged for performance is CADD [22], which estimates the deleteriousness of SNVs and short insertions and deletions (InDels). These methods can introduce ascertainment bias for variants that are under high evolutionary pressure (such as nonsense and splicing variants) even though these variants can also be observed in healthy populations, and they can neglect rare and recent variants that have not undergone purifying selection but are still found to contribute to diseases [23].

New computational prediction methods need to be assessed for their ability to reduce the number of variants that require time-consuming expert evaluation as this is currently a bottleneck in the diagnostic pipeline. With hundreds to thousands of non-pathogenic variants identified in a typical patient with a rare genetic disorder, it is important to restrict the false-positive rate of computational prediction methods, i.e., reduce the number of neutral variants falsely reported as pathogenic. However, new methods are not often evaluated for their ability to recognize neutral variants. Indeed, a recent review [24] found that commonly used variant interpretation tools may incorrectly predict a third of the common variations found in the Exome Aggregation Consortium (ExAC) to be harmful. We speculate that this may be explained by the bias in training data selection because the neutral set used in different tools can be biased towards common neutral variants [15, 25, 26], which in practice means that the pathogenicity of rare and ultra-rare variants cannot be accurately estimated. Therefore, it is important to avoid bias in data selection and evaluate false-positive rate of the prediction methods in clinical setting where rare and ultra-rare neutral variants are frequently encountered using neutral benchmark datasets [27, 28] and clinical data.

The challenge for rare disease research and diagnostics is thus to find robust classification algorithms that perform well for all the different types of variants and allele frequencies. To meet this challenge, we developed CAPICE, a new method for Consequence-Agnostic prediction of Pathogenicity Interpretation of Clinical Exome variations. CAPICE overcomes limitations common in current predictors by training a sophisticated machine-learning model that targets (non-)pathogenicity using a specifically prepared, high confidence and pathogenicity versus benign balanced training dataset, and using many existing genomic annotations across the entire genome (the same features that were used to produce CADD). In high-quality benchmark sets, CAPICE thus outperforms existing methods in distinguishing pathogenic variants from neutral variants, irrespective of their different molecular consequences and allele frequency. To our knowledge, CAPICE is also the first and only variant prioritization method that targets pathogenicity prediction of all types of SNVs and InDels, irrespective of consequence type.

Below we describe the results of our performance evaluations, discuss features and limitations of our methodology, and provide extensive details on the materials and methods used, concluding that CAPICE thus offers high accuracy pathogenicity classification across all consequence types and allele frequencies, outperforming all next-best variant classification methods. To make CAPICE easy to access, we have developed CAPICE as both a command-line tool and a web-app and released it with pre-computed scores available as ready-to-use annotation files.

## Methods

**Fig. 1:**
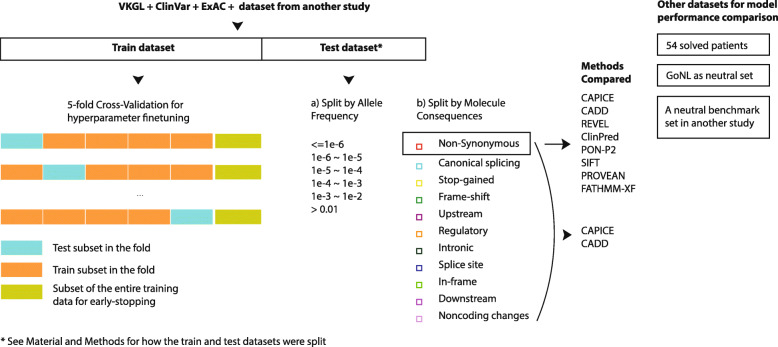
An overview of the study setup

The flowchart of this study is shown in Fig. 1. Briefly, we collected variant annotation and classification data from multiple sources and used gradient boosting on decision trees to train our pathogenic variant prioritizing model with the same set of features used to build CADD scores. We subsequently evaluated our model in a balanced benchmark dataset and examined its performance for subgroups of variants in that benchmark dataset. Additionally, we tested our model on two benign benchmark datasets. To demonstrate its application in clinic, we applied our model to data from 54 solved patients and compared its prioritization results against those obtained by CADD for the same data.

### Data collection and selection

An overview of the training and benchmark datasets can be found in Table 1. Training and benchmark data on neutral and pathogenic variants were derived from vcf files from the ClinVar database [17], dated 02 January 2019; from the VKGL data share consortium [30]; from the GoNL data [31]; and from data used in a previous study [29]. From the ClinVar dataset, we collected variants reported by one or more submitters to have clear clinical significance, including pathogenic and likely pathogenic variants and neutral and likely neutral variants. From the VKGL data consortium, we collected variants with clear classifications, either (Likely) Pathogenic or (Likely) Benign, with support from one or more laboratories. The neutral variants from previous research developing the GAVIN tool [29] were mainly collected from ExAC without posing a constraint on allele frequency. We also obtained two neutral benchmark datasets from a benchmark study by [24] and the GoNL project.

**Table 1:** Data source for the variants and pathogenicity interpretation

Data name	Data source	Number of pathogenic variants	Number of neutral variants
Training dataset	ClinVar (≥ 1 stars)	10,370	14,954
	VKGL (≥ 1 lab support)	581	11,129
	van der Velde et al. [29]	30,187	274,112
	Total *	**40,681**	**293,920**
Benchmark dataset	ClinVar (≥ 2 stars)	5421	20
	VKGL (≥ 2 lab support)	187	11
	ExAC	0	5392
	**Total**	**5421**	**5421**
Benign Benchmark dataset 1	Niroula et al. [24]	0	60,699
Benign Benchmark dataset 2	GoNL	0	14,426,914

*The total numbers of variants are smaller or equal to the sum of variants from all data sources due to the removal of duplicated variants

In our data selection step, we removed duplicate variants located in unique chromosomal positions and those with inconsistent pathogenicity classification across the different databases. To reduce potential variants in general population datasets from carriers, we excluded variants observed in dominant genes using inheritance modes of each gene retrieved from the Clinical Genome Database dated 28 February 2019 [32].

In total, we collected 80k pathogenic variants and 450k putative neutral variants, and the training and benchmark datasets can be found online. After the initial cleaning step described above, we built a training dataset for model construction and a benchmark dataset that we left out of the training procedures so it could be used for performance evaluation later on.

### Construction of the benchmark and training sets

To build a benchmark dataset for performance evaluation that was fully independent of model construction procedures, we selected high-confidence pathogenic variants from the ClinVar and VKGL databases and neutral variants from both the curated databases ClinVar and VKGL, and from the population database ExAC. The high-confidence pathogenic variants are ClinVar variants with a review status of “two or more submitters providing assertion criteria provided the same interpretation (criteria provided, multiple submitters, no conflicts),” “review by expert panel,” and “practice guideline” in ClinVar database and VKGL variants that are reported by one of more laboratories without conflicting interpretation in VKGL database. From the pathogenic variants that passed these criteria, we then randomly selected 50% to add into the benchmark dataset, which resulted in 5421 pathogenic variants. During our analysis, we found that variants’ molecular effects and allele frequency influence the model performance. Therefore, to enable unbiased comparison, we created benchmark datasets with equal proportions of pathogenic and neutral variants for each type of molecular consequences, with the additional requirement that the pathogenic and neutral variants share similar distributions in allele frequency. An overview of the allele frequency distribution of the pathogenic and neutral variants for each type of molecular effects is in Additional File 1: Fig. S1.

In total, our benchmark set contained 10,842 variants and our training set contained 334,601 variants.

For our training dataset, we combined the collected high-confidence variants that are not present in the benchmark datasets, the low-confidence variants in ClinVar and VKGL, the variants from [29], and the neutral variants from ExAC that are not present in the benchmark dataset. The training set had 32,783 high confidence variants and 301,819 lower confidence variants. The high-confidence training variants were 12,646 pathogenic variants and 20,137 neutral variants. The lower confidence variants were 28,035 pathogenic variants and 273,783 neutral variants.

The two neutral benchmark datasets are those taken from a previous benchmark study and the GoNL dataset. The previous benchmark study [24] selected neutral variants from the ExAC dataset and only included common variants with allele frequencies between 1 and 25%. For this dataset, we removed variants seen in the training set. In total, there were 60,699 neutral variants in our benchmark dataset. To build the neutral benchmark dataset from GoNL data, we selected all the variants that passed the assessment of the genotype variant calling quality. Concretely, we selected all variants with a “PASS” recorded in the “QUAL” column in the VCF files downloaded from the data source. Then we calculated the variants’ allele frequency within the GoNL population and selected those with an allele frequency < 1% that had not been seen in the training set. In total, there were 14,426,914 variants involved (Additional File 1: Table S2).

### Data annotation and preprocessing

The collected variants in both the training and test datasets were annotated using CADD web service v1.4, which consists of 92 different features from VEP (version 90.5) [33] and epigenetic information from ENCODE [34] and the NIH RoadMap project [35]. A detailed explanation of these features can be found in Kircher et al.’s [21] CADD paper. For each of the 11 categorical features, we selected up to five top levels to avoid introducing excessive sparsity, which could be computationally expensive, and used one-hot encoding before feeding the data into the model training procedures [36]. For the 81 numerical variables, we imputed each feature using the imputation value recommended by Kircher et al. [21]. The allele frequency in the population was annotated using the vcfTool [37] from GnomAD r2.0.1 [38]. We assigned variants not found in the GnomAD database an allele frequency of 0.

### Model construction and training

We trained a gradient-boosting tree model using the XGBoost (version 0.72) Python package. The hyper-parameters, n_estimators, max_depth, and learning_rate were selected by 5-fold cross-validation using the RandomSearchCV function provided by the scikit-learn (version 0.19.1) Python package. Within each training fold, we used an early stopping criteria of 15 iterations. We then used the model trained with the best set of hyper-parameters (0.1 for learning_rate, 15 for max_depth, and 422 for n_estimators) for performance measurement. For fitting the model, we also used the sample weight assigned to each variant. The sample weight is a score ranging from 0 to 1 that reflects the confidence level of the trustworthiness of the pathogenicity status of that variant. High-confidence variant, as described previously, are given a sample weight of 1, and the low-confidence variants were given a lower sample weight of 0.8. A variant with a high sample weight will thus contribute more to the loss function used in the training procedure [36]. To test the assigned sample weights, we used the best set of parameters returned from the previous fine-tuning process and tried three different conditions in which we set the sample weights of the lower confidence variants to 0, 0.8, and 1. We then selected the model with the highest AUC value for the cross-validation dataset.

### Threshold selection strategies

For comparing the false-positive rate in the neutral benchmark dataset and comparing the classification results, we tested different threshold-selection strategies for both CAPICE and CADD. For CAPICE, we obtained the threshold from the training dataset that results in a recall value within 0.94–0.96. To calculate the threshold, we searched for all possible threshold value from 0 to 1 and selected the first threshold for which the resulting recall value fall between 0.94 and 0.96. This method resulted in a general threshold of 0.02. For CADD, we tested two different threshold-selection methods. The first threshold was a default value of 20. The second method used GAVIN [29] to provide gene-specific thresholds. For other machine learning methods that returned a pathogenicity score ranging from 0 to 1, and no recommended threshold was given in the original paper, we selected a default value of 0.5. This includes the following methods: REVEL, ClinPred, SIFT, and FATHMM-XF. For PROVEAN, we used a default score of − 2.5 as the threshold.

### Evaluation metrics

For model performance comparison, we used receiver operating characteristic (ROC) curve, AUC value [39], and measurements in the confusion matrix together with the threshold-selection strategies mentioned above. For measuring model performance in the neutral benchmark dataset, we examined the false-positive rate. The false-positive rate is the number of true neutral variants but predicted as pathogenic divided by the number of true neutral variants. To evaluate the robustness of the model predictions, we performed bootstrap on the benchmark dataset for standard deviation measurement for 100 repetitions, with the same sample size of the benchmark dataset for each repetition [40].

To evaluate performance in solved patients, we used the previously diagnosed patients with clear record of the disease-causing variant from University Medical Center in Groningen. A description of the solved patients can be found in [41]. For examining CAPICE’s performance, we first eliminated all variants with an allele frequency > 10% and then predicted the pathogenicity for the remaining variants. Subsequently, we sorted the variants of each individual by their pathogenicity score assigned by the respective predictors and used the ranking of the disease-causing variant found within that individual as the measurement.

## Results

CAPICE is a general prediction method that provides pathogenicity estimations for SNVs and InDels across different molecular consequences. We used the same set of features that the CADD score was built upon and trained a gradient-boosting tree model directly on the variant pathogenicity. In our performance comparison, we compared CAPICE against recently published methods and those that showed best performance in benchmark studies. Below we report performance analysis of CAPICE using gold standard benchmark sets, analysis of the classification consistency of CAPICE across different allele frequency ranges and across different types of variants, and a small practical evaluation where we applied CAPICE to a set of patient exomes.

### CAPICE outperforms the best current prediction methods

**Fig. 2:**
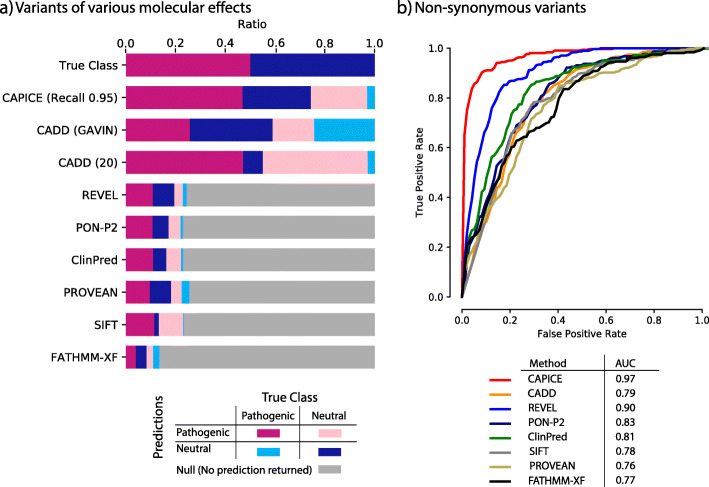
CAPICE outperforms other predictors in discriminating pathogenic variants and neutral variants. **a** True/false classification for all predictors tested against the full benchmark set that contains all types of variants. Top bar shows the breakdown of the test set. Other bars show the classification performance for each method. Purple blocks represent correct classification of pathogenic variants. Dark-blue blocks represent neutral variants. Pink and light-blue blocks denote false classifications. Gray blocks represent variants that were not classified by the predictor tested. Threshold selection methods are described in the “Methods” section. **b** Receiver operating characteristic (ROC) curves of CAPICE with AUC values for a subset of the benchmark data that only contains non-synonymous variants (the ROC curve for the full dataset can be found in Additional File 1: Fig. S2). Each ROC curve is for a subset of variants displaying a specific molecular consequence. AUC values for the different methods are listed in the figure legend

In our benchmark datasets, CAPICE performs as well or better than other current prediction methods across all categories (Fig. 2, Additional File 1: Fig. S2, Fig. S3 Table S2, Table S3). Because most prediction methods are built specifically for non-synonymous variants, we performed the comparison for both the full dataset and the non-synonymous subset. For the case where a tool was not able to provide a prediction, we marked it as “No prediction returned.” We also examined the robustness of CAPICE’s performance for rare and ultra-rare variants and variants that lead to different consequences.

For the full data, CAPICE outperformed CADD, the mostly used “general” prediction method, and achieved an area under the receiver operating characteristic curve (AUC) of 0.89 as compared to 0.53 for CADD (shown in Additional File 1: Fig. S2). For the non-synonymous subset, CAPICE outperformed all the other prediction methods and achieved an AUC of 0.97 (shown in Fig. 2b). The majority of other methods we examined are built specifically for non-synonymous variants, with the exception of FATHMM-XF, which was developed for point mutations. For the non-synonymous subset, REVEL, which was built for rare variants, produced the second best result and achieved an AUC of 0.90.

To assess the impact of this difference in practice, we assumed a clinical setting with the aim to recognize 95% of the pathogenic variants (which is a very high standard in current practice). When using a threshold of 0.02 on CAPICE classification score, CAPICE correctly recognized 95% of pathogenic variants in the full test dataset and wrongly classified 50% of the neutral variants as pathogenic—which was the lowest number of misclassified variants among all the predictors we tested. In contrast, CADD with a score threshold of 20 achieved a comparable recall of 94%, but wrongly classified 85% of neutral variants as pathogenic. When using gene-specific CADD score thresholds based on the GAVIN method [29], the performance of CADD was better but still much worse than CAPICE. All other tested methods could give predictions less than 30% of the full dataset.

We also examined how well the prediction methods can recognize neutral variants in two neutral benchmark datasets. For both datasets, CAPICE’s performance was comparable to or better than the current best prediction methods (Additional File 1: Table S2, Table S3).

### CAPICE outperforms other current predictors for rare and ultra-rare variants

**Fig. 3:**
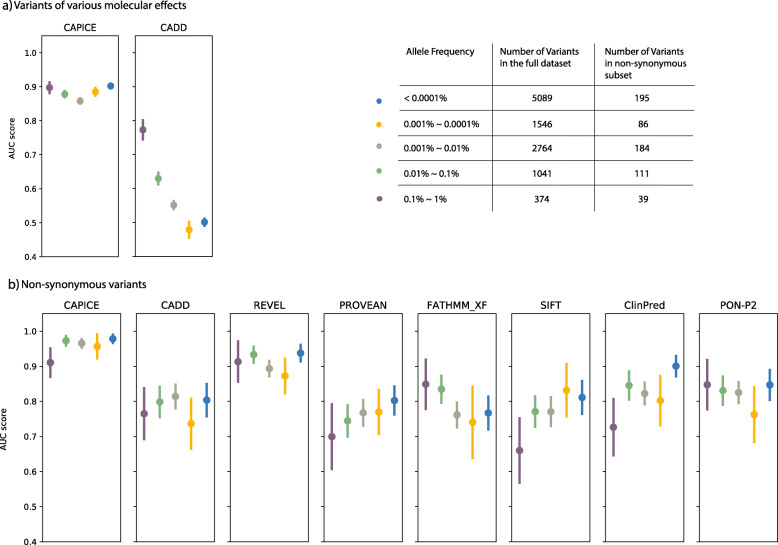
Performance comparison for rare and ultra-rare variants for **a** variants with different molecular consequences and **b** in the missense subset. Each dot represents the mean AUC value with standard deviation

CAPICE performs consistently across different allele frequencies and especially well for rare and ultra-rare variants. Here we repeated the evaluation strategy for the same benchmark dataset grouped into five allele frequency bins (For the full benchmark dataset, CAPICE performed consistently above 0.85 of AUC for variants with an allele frequency < 1%, while the performance of CADD version 1.4 [42], the current best method for indicating the pathogenicity of variants throughout the genome compared to LINSIGHT [43], EIGEN [44], and DeepSEA [45] dropped significantly in case of rare variants (Fig. 3a). For the non-synonymous subset, CAPICE consistently performed better or comparably to the next-best method, REVEL, for variants within different allele frequency ranges, and better than all other methods (Fig. 3b).

For common variants (defined here as having an allele frequency > 1%), the number of available pathogenic variants was too small (14 pathogenic variants) to get an accurate and robust performance measurement.

### CAPICE shows consistent prediction performance for different types of variants

**Fig. 4:**
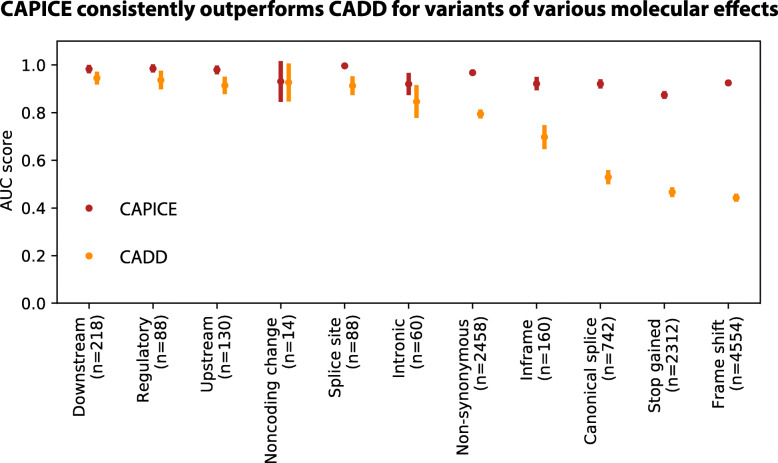
Performance comparison of CAPICE and CADD for variants of different molecular consequences

CAPICE outperforms the best current computational prediction methods for variants that cause different molecular consequences (Fig. 4 and Additional File 1: Fig. S2). For these variants, CAPICE has an AUC of 0.92 for canonical splicing variants and an AUC of 0.97 for non-synonymous variants in the independent test dataset. Compared to CADD, CAPICE performs significantly better for multiple types of variants, particularly canonical splicing, stop-gained and frameshift variants.

### CAPICE performance in a clinical setting

**Fig. 5:**
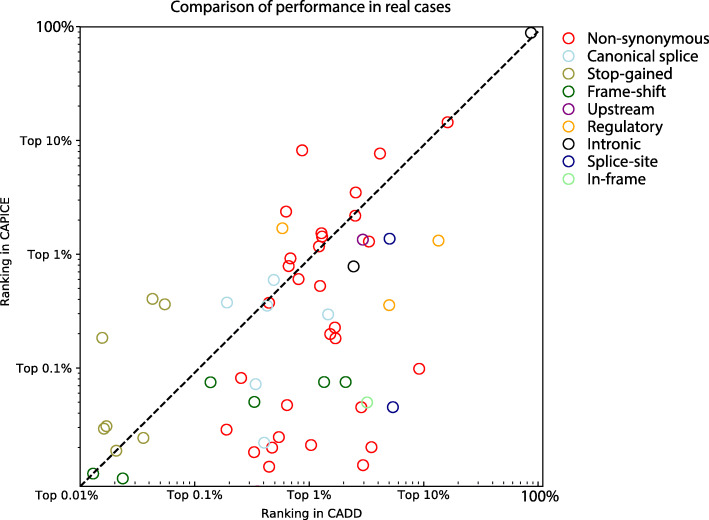
Performance comparison in real cases. In total, 54 patients and 58 variants were included. Each variant is reported as the diagnosis for that patient. Each dot in the plot shows a variant. The color of the dot represents the molecular effect predicted by VEP

To make our first assessment of clinical utility, we used whole-exome sequencing data from 54 solved patients from our diagnostics department and compared the ranking of the disease-causing variant with scores from CADD and CAPICE (Fig. 5). We did not compare to REVEL, the second-best method from our previous evaluation, because a specific method for non-synonymous variants can miss variants of other molecular effects. A description of the solved patients’ can be found in [41]. For each disease-causing variant discovered in that patient, we compared the performance of CAPICE and CADD by comparing the ranking of the particular variant among all variants observed within that patient. For 83% of the cases, CAPICE can prioritize the disease-causing variant within the 1% of the total variants observed in whole exome sequencing experiment, while CADD achieves the 1% performance for only 60% of the cases. Consistent with results described in previous sections that CAPICE achieves better AUC value for frameshift variants, CAPICE performed better for all cases with a disease-causing variant of frameshift effect.

## Discussion

We have implemented a supervised machine-learning approach called CAPICE to prioritize pathogenic SNVs and InDels for genomic diagnostics. CAPICE overcomes the limitations of existing methods, which either give predictions for a particular type of variants or show moderate performance because they are built for general purposes. We showed in multiple benchmark datasets, either derived from public databases or real patient cases, that CAPICE outperforms the current best method for rare and ultra-rare variants with various molecular effects. To compare CAPICE’s performance with existing methods, we chose only recently published methods that have consistently performed well in various independent benchmark studies.

In this study, we used the same set of features as CADD used for constructing their score but trained the model directly on pathogenicity. The features enabled CAPICE to make predictions for variants of various molecular effects. Its focus on pathogenicity helped CAPICE to overcome the challenges faced by CADD in predicting pathogenicity [46] in the clinic. As a result, CAPICE gives significantly better prediction for rare variants, and various types of variants, in particular, frameshift, splicing, and stop-gained variants. We also observed that most current predictors have problems classifying rare and ultra-rare variants, with the exception for REVEL, an ensemble method that targets rare variants. We thus adopted the same strategy as REVEL by including rare variants when training CAPICE and thereby obtained a comparable performance to that of REVEL for missense rare variants and significantly better results than all the other methods tested for ultra-rare variants. Tree-based machine learning models have shown superior performance in classifying pathogenic and benign variants. For instance, REVEL uses a random forest and ClinPred uses a combined score from a random forest and gradient-boosting trees. We compared the performance of both methods as shown in Additional File 1: Fig. S6 and chose gradient boosting for its better performance. We also show that the choice of training dataset for pathogenic variants, e.g., ClinVar or VKGL, does not greatly influence model performance (Additional File 1: Fig. S4).

We made full use of the large amount of data generated by other researchers. The evidence for a variant’s clinical relevance reported in public databases such as ClinVar can be conflicting or outdated [47]. The star system used in ClinVar review status [48] serves as a good quality check for estimating the trustworthiness of the reported pathogenicity, and this quality estimation is used by many researchers as a selection criteria for constructing or evaluating variant prioritization methods [15, 49]. However, this method of data selection can introduce biases and waste potentially important information. In particular, neutral variants can be enriched for common ones. These common variants can be easily filtered out in a diagnostic pipeline using a general cut-off or expected carrier prevalence for specific diseases [50]. Using such a biased dataset could however lead to a biased model or an overly optimistic performance estimation. When training CAPICE, we did not exclude lower-quality data, but rather assigned it a lower sample weight during model training. We also showed that training on high-quality data does not improve model performance (Additional File 1: Fig. S5). This strategy overcame the data selection bias mentioned above and led to a model with equally good performance for both rare and ultra-rare variants. When testing CAPICE, we selected only high-quality data for the pathogenic set. For the neutral set, we included rare and ultra-rare variants for all the types of variations found in general population studies (after filtering for known pathogenic variations and inheritance mode). This allowed us to avoid the bias discussed above.

Current variant prioritization methods, including ours, often neglect context information about a patient such as phenotype, family history and the cell-type associated with specific diseases. Moreover, the methods developed are often evaluated in a stand-alone manner, and their associations with other steps in a genome diagnostic pipeline are not often investigated. In this study, we have only shown preliminary evaluation results using solved patient data. In future studies, we hope to include context information to further improve CAPICE’s predictive power. We also believe that the model’s performance needs to be discussed in a broader context that includes gene prioritization and mutational burden-testing.

## Conclusions

We have developed CAPICE, an ensemble method for prioritizing pathogenic variants in clinical exomes for Mendelian disorders, including SNVs and InDels. CAPICE outperforms all other existing methods, and it is our hope that it greatly benefits rare disease research and patients worldwide. By re-using the CADD features, but training a machine-learning model on variants’ pathogenicity, CAPICE consistently outperforms other methods in our benchmark datasets for variants with various molecular effect and allele frequency. Additionally, we demonstrate that predictions made using CAPICE scores produce many fewer false positives than predictions made based on CADD scores. To enable its integration into automated and manual diagnostic pipelines, CAPICE is available as a free and open source software command-line tool from https://github.com/molgenis/capice and as a web-app at https://molgenis.org/capice. Pre-computed scores are available as a download at https://zenodo.org/record/3928295.

## Availability and requirements

Project name: CAPICE.

Project home page: https://github.com/molgenis/capice

Demo site for the web service: https://molgenis.org/capice

Operating system(s): Platform independent.

Programming language: Python 3.6.

License: GNU Lesser General Public License v3.0.

Any restrictions to use by non-academics: none.

Resources used in this study:

CADD: https://cadd.gs.washington.edu/score

REVEL: https://sites.google.com/site/revelgenomics/

PON-P2: http://structure.bmc.lu.se/PON-P2/

ClinPred: https://sites.google.com/site/clinpred/

PROVEAN and SIFT: http://provean.jcvi.org/genome_submit_2.php?species=human

GAVIN: https://molgenis.org/gavin

FATHMM-XF: http://fathmm.biocompute.org.uk/fathmm-xf/

ClinVar: https://ftp.ncbi.nlm.nih.gov/pub/clinvar/vcf_GRCh37/archive_2.0/2019/clinvar_20190731.vcf.gz

GoNL: http://molgenis26.gcc.rug.nl/downloads/gonl_public/releases/release2_noContam_noChildren_with_AN_AC_stripped.tgz

## Supplementary information

**Additional file 1.**

## Data Availability

The whole exome sequencing data for the 54 patients used in this study was used in a previously published study [41], and while patients allow anonymous use of their data for research purposes, explicit written informed consent to publish was not obtained. Thus, this data cannot be shared due to patient privacy concerns. Training and testing data with label and predictions from CAPICE and tested predictors and the pre-computed scores for all possible SNVs and InDels are available online at Zenodo [51]: https://zenodo.org/record/3928295 and at GitHub: https://github.com/molgenis/capice.
