# Supplementary material for: CAPICE: a computational method for Consequence-Agnostic Pathogenicity Interpretation of Clinical Exome variations
Source: Genome Med. 2020 Aug 24;12:75. doi: 10.1186/s13073-020-00775-w (PMC7446154; doi:10.1186/s13073-020-00775-w)
Supplement: Supplementary file 1 — Additional file 1. [file 13073_2020_775_MOESM1_ESM.pdf]

CAPICE: a computational method for Consequence-Agnostic Pathogenicity Interpretation of Clinical Exome variations

Shuang Li, K. Joeri van der Velde, Dick de Ridder, Aalt D.J. van Dijk, Dimitrios Soudis, Leslie R. Zwerwer, Patrick Deelen, Dennis Hendriksen, Bart Charbon, Marielle E. van Gijn, Kristin Abbott, Birgit Sikkema-Raddatz, Cleo C. van Diemen, Wilhelmina S. Kerstjens-Frederikse, Richard J. Sinke, Morris A. Swertz

Supplementary Figures

Fig. S1: The balanced benchmark dataset-- 2

Fig. S2: Model performance between CAPICE and CADD in terms of ROC curves and AUC values for variants with different molecular functions.--- 3

Fig. S3: Model performance among CAPICE and other methods for the full dataset and subsets separated by allele frequency-- 4

Fig. S4: Model performance on the ClinVar and VKGL test dataset when the model is trained on either a) ClinVar variants or b) VKGL variants. -- 5

Fig. S5: Model performance on the benchmark dataset for training data selection. ------------- 6

Fig. S6: Model selection --- 6

Supplementary Tables

Table S1: Description of all methods tested in the study -- 7

Table S2: CAPICE and CADD false positive rates in the neutral benchmark dataset------------ 8

Table S3: CAPICE and CADD false positive rates in the GoNL dataset---------------------------- 9

Supplementary Figures

Fig. S1: The balanced benchmark dataset

As described in the Methods section, we created a balanced benchmark dataset that is equally distributed in terms of a) the number of pathogenic and putatively neutral variants and b) allele frequency distribution for different molecular consequences between the pathogenic and neutral variants.

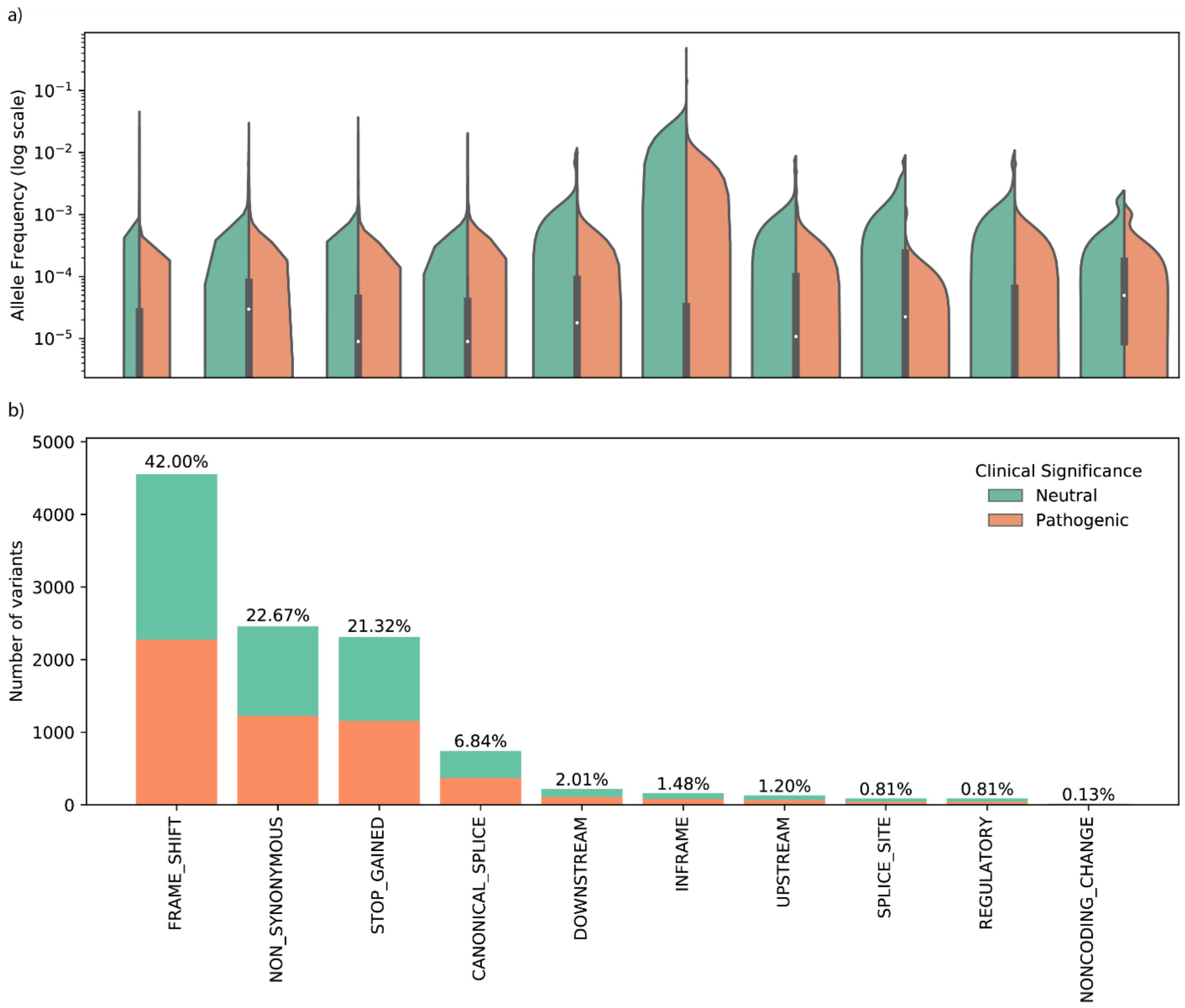

Fig. S2: Model performance between CAPICE and CADD in terms of ROC curves and AUC values for variants with different molecular functions.

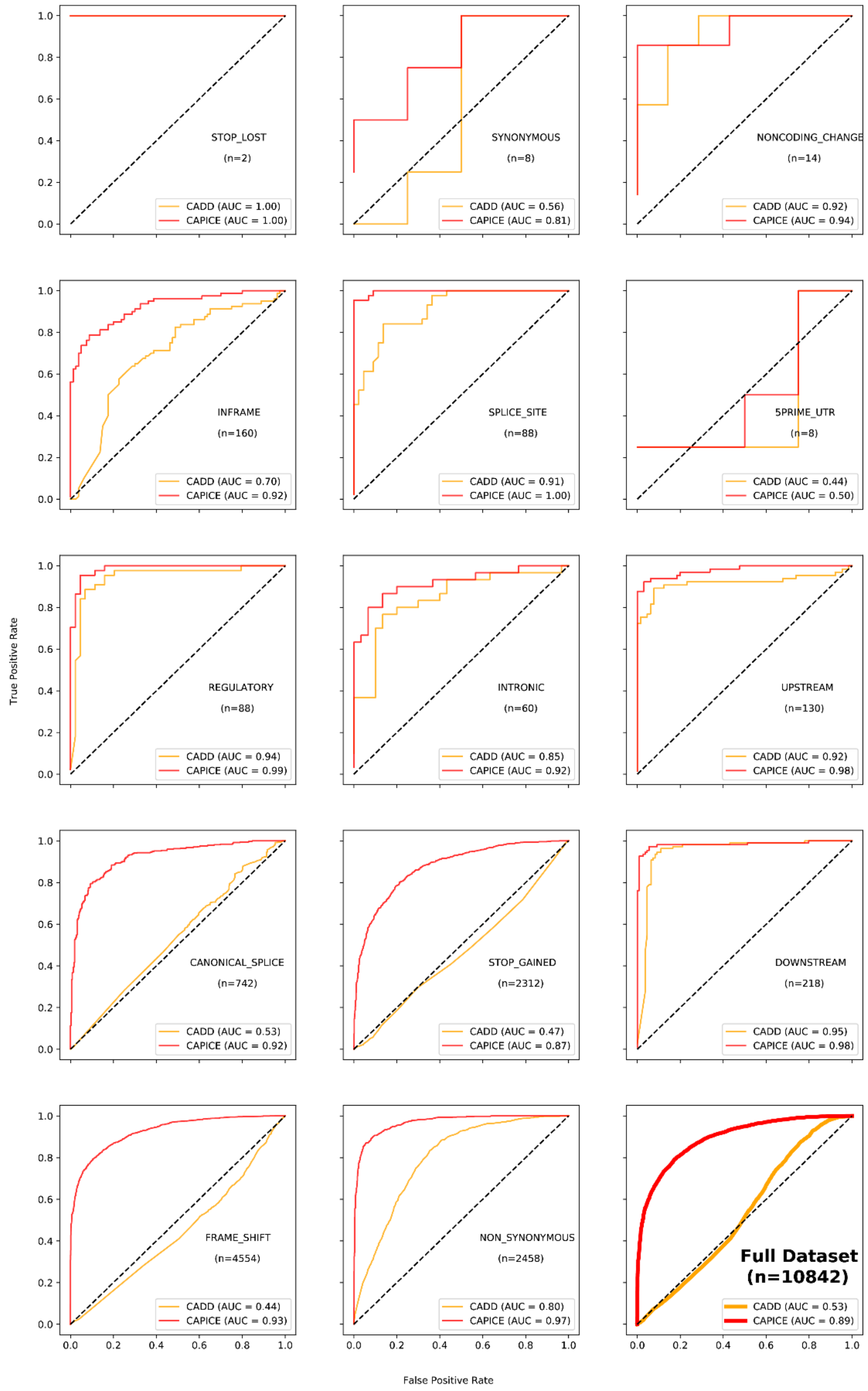

Fig. S3: Model performance among CAPICE and other methods for the full dataset and subsets separated by allele frequency

Below we show the AUC value and the ROC curves for the a) full dataset and b) missense subset for rare and ultra-rare variants defined as variants with allele frequency between 0.01% and 0.1% and variants with allele frequency <0.01%, respectively.

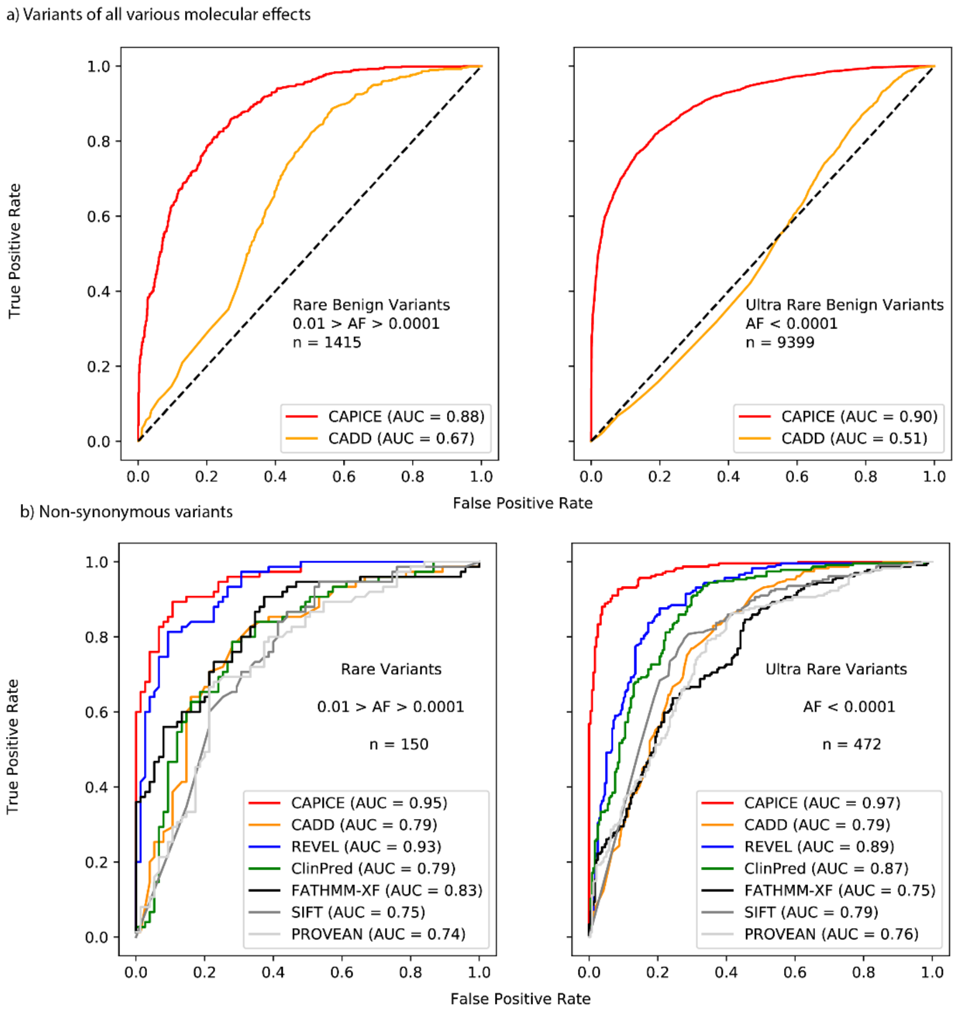

Fig. S4: Model performance on the ClinVar and VKGL test dataset when the model is trained on either a) ClinVar variants or b) VKGL variants.

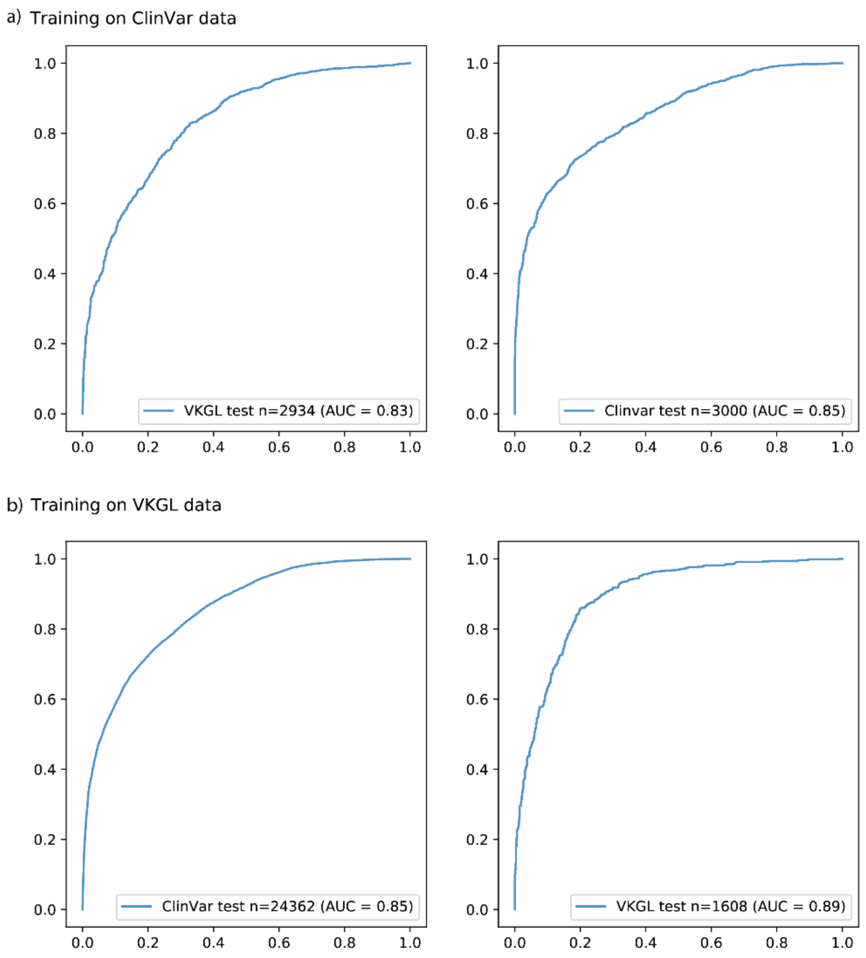

Fig. S5: Model performance on the benchmark dataset for training data selection.

To elaborate on our reasoning on training data selection, we compared the following training data selection in terms of the AUC value and ROC curves: a) curated variants from ClinVar with ≥ 2 stars as the ClinVar review status b) all variants in our training dataset or c) randomly sampled variants from our training dataset with the same number as the curated variants.

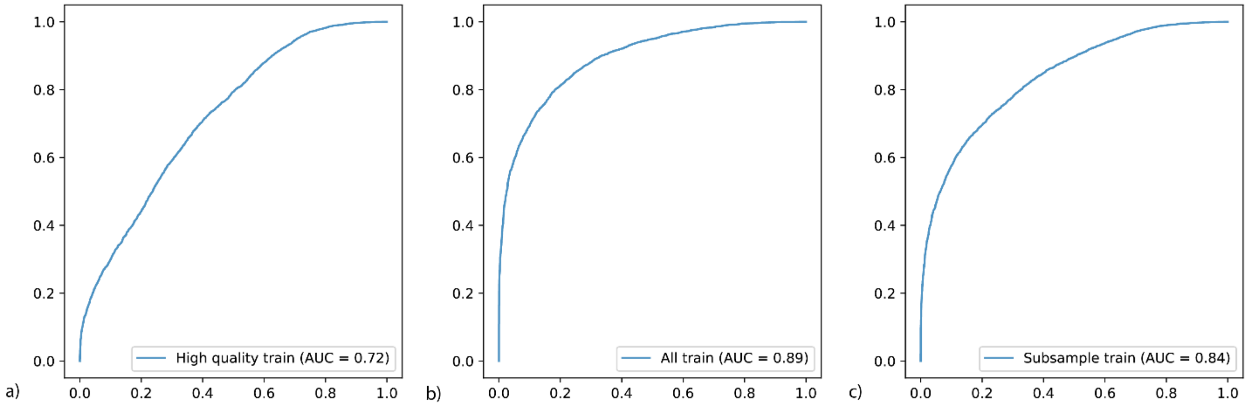

Fig. S6: Model selection

Below we elaborated our reasoning on model selection and showed the ROC curves and AUC values on the benchmark dataset between a) a random forest model and b) gradient boosting on decision trees.

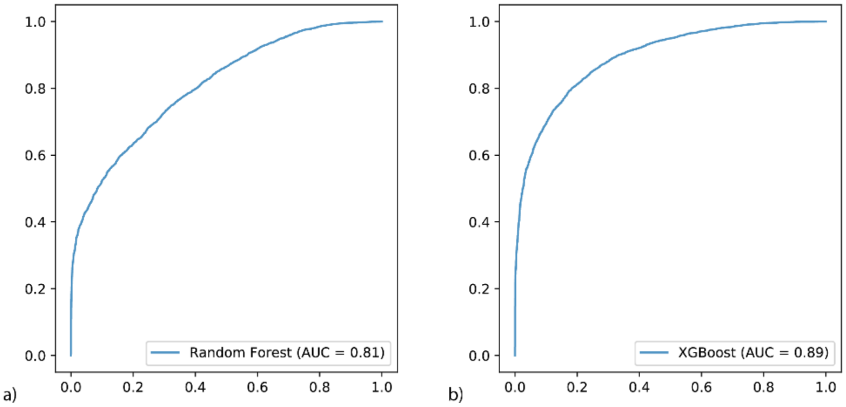

Supplementary Tables

Table S1: Description of all methods tested in the study

Method	Application	Link for web resources
CADD	Estimating the relative pathogenicity of SNVs and InDels	https://cadd.gs.washington.edu/
REVEL	Predicting the pathogenicity of rare missense variants	https://sites.google.com/site/revelgenomics/
ClinPred	Predicting the pathogenicity of missense variants	https://sites.google.com/site/clinpred/
PON-P2	Predicting the pathogenicity of missense variants	http://structure.bmc.lu.se/PON-P2/
SIFT	Predicting missense variants effects on protein function	http://provean.jcvi.org/genome_submit_2.php?species=human
PROVEAN	Predicting missense variants and InDels' effects on protein function	http://provean.jcvi.org/genome_submit_2.php?species=human
FATHMM-XF	Predicting pathogenicity of point mutations	http://fathmm.biocompute.org.uk/fathmm-xf/

Table S2: CAPICE and CADD false positive rates in the neutral benchmark dataset

Molecular Consequence	CADD (20)	CAPICE (Threshold with recall being 0.95)	Number of Variants
NON_SYNONYMOUS	0.37	0.07	50181
DOWNSTREAM	0.36	0.11	12121
REGULATORY	0.35	0.12	11317
UPSTREAM	0.35	0.1	10959
INTRONIC	0.25	0.06	10910
NONCODING_CHANGE	0.34	0.06	1202
3PRIME_UTR	0.23	0.03	902
SYNONYMOUS	0.07	0.08	456
5PRIME_UTR	0.19	0.03	295
SPLICE_SITE	0.15	0.08	137
CANONICAL_SPLICE	0.6	0.4	10
STOP_GAINED	1	0.22	9

Table S3: CAPICE and CADD false positive rates in the GoNL dataset

Molecular Consequence	CADD (20)	CAPICE (Threshold with recall being 0.95)	Number of Variants
INTRONIC	0.00	0.00	6483483
INTERGENIC	0.00	0.00	4399474
DOWNSTREAM	0.01	0.00	1205398
UPSTREAM	0.01	0.00	1174342
REGULATORY	0.01	0.01	808360
NONCODING_CHANGE	0.01	0.00	115099
3PRIME_UTR	0.02	0.00	108194
NON_SYNONYMOUS	0.57	0.19	67013
SYNONYMOUS	0.02	0.08	36177
5PRIME_UTR	0.03	0.01	14526
SPLICE_SITE	0.05	0.08	10352
CANONICAL_SPLICE	0.58	0.54	1545
STOP_GAINED	1.00	0.74	1647
FRAME_SHIFT	0.88	0.9	821
INFRAME	0.37	0.63	415
STOP_LOST	0.06	0.37	68
